# A Multiscale Model for Virus Capsid Dynamics

**DOI:** 10.1155/2010/308627

**Published:** 2010-03-09

**Authors:** Changjun Chen, Rishu Saxena, Guo-Wei Wei

**Affiliations:** ^1^Department of Mathematics, Michigan State University, East Lansing, MI 48824, USA; ^2^Department of Electrical and Computer Engineering, Michigan State University, East Lansing, MI 48824, USA

## Abstract

Viruses are infectious agents that can cause epidemics and pandemics. The understanding of virus formation, evolution, stability, and interaction with host cells is of great importance to the scientific community and public health. Typically, a virus complex in association with its aquatic environment poses a fabulous challenge to theoretical description and prediction. In this work, we propose a differential geometry-based multiscale paradigm to model complex biomolecule systems. In our approach, the differential geometry theory of surfaces and geometric measure theory are employed as a natural means to couple the macroscopic continuum domain of the fluid mechanical description of the aquatic environment from the microscopic discrete domain of the atomistic description of the biomolecule. A multiscale action functional is constructed as a unified framework to derive the governing equations for the dynamics of different scales. We show that the classical Navier-Stokes equation for the fluid dynamics and Newton's equation for the molecular dynamics can be derived from the least action principle. These equations are coupled through the continuum-discrete interface whose dynamics is governed by potential driven geometric flows.

## 1. Introduction

Viruses are omnipresent infectious agents that are about 100 times smaller than bacteria. Unlike bacteria, viruses are not able to grow or reproduce outside a host cell [1–4]. There are more than 5000 types of known viruses. Viruses have a known history of causing epidemics and pandemics. About 70% of native Americans were killed by foreign diseases after the arrival of Columbus in the Americas. The Spanish flu pandemic lasted from 1918 to 1919 and killed about 100 million people, or 5% of the world's population in 1918. AIDS, a disease due to HIV virus, has killed more than 25 million people since it was first recognized on June 5, 1981. There are about 39 million people living with HIV viruses worldwide nowadays. Virus infection processes or virus life cycles differ greatly among species but there are six basic stages: [1–4] (1) selective attachment due to the interaction, binding and/or fusion between viral capsid surface and specific receptors on the host cellular surface, (2) penetration of a virus into a host cell through membrane fusion or receptor-mediated endocytosis, (3) viral genomic nucleic acid releasing in the host cell due to viral capsid degradation by viral enzymes or host enzymes, (4) virus replication and assembly in the host cell, (5) Posttranslational modification of the viral proteins; and finally, (6) virus releasing from the host cell. For some viruses, such as HIV, the order of stages (5) and (6) is reversed. Body uses two defense mechanisms, innate immune system and cell-mediated immunity to defend host from infection by viruses or other organisms. The innate immune system terminates the virus replication in the host cell by degrading or inhibiting the virus genetic material, DNA or RNA through antibodies or other virus DNA/RNA binding molecules. In the cell-mediated immunity, killer cells known as T cells destroy the infected host cell and its close neighbors by recognizing the viral protein displayed on the cellular surface.

Recent advances in structural biology and microbiology have led to a rapidly growing body of virus structural data [5–7]. A striking feature of virus data is that they are excessively large—a virus complex may involve tens of millions atoms, with detailed information on atomic coordinates, types, and radii. Most virus structural data are collected via X-ray diffraction (X-ray), cryo-electron microscopy (cryoEM) [8], fiber diffraction, and nuclear magnetic resonance (NMR) techniques. There are a few major virus morphologies: spherical type, helical type, dihedral type, viral envelope type, and complex type. Most animal viruses are of spherical morphology with icosahedral symmetry [6]. Most virus structure information can be obtained from the Protein Data Bank (PDB; http://www.rcsb.org/pdb/home/home.do), the Virus Particle Explorer database [7] (VIPERdb; http://viperdb.scripps.edu/), and the Protein Quaternary Structure server (PQS; http://pqs.ebi.ac.uk/).

Currently, the prevention and control of epidemics and pandemics caused by infective viruses, such as H1N1, HIV, SARS, and bird flu are of paramount importance. As an infection starts with the surface attachment between a virus and a host cell, it is important to construct and visualize the surface topology and morphology of viruses in order to understand the surface attachment and further interaction. This information is also crucial to the understanding of the molecular mechanism that gives rise to the assembly of virus capsids and DNA or RNA packaging. Computer-based visualization is able to represent results of explorations in an easy-to-comprehend form and to facilitate convenient information retrieval. Currently, visualization tools are often developed in close conjunction with imaging, data registration, simulation and/or surface construction. Virus visualization plays a unique role in the understanding of virus infection processes, such as, virus attachment of a host cell, binding and fusion between a virus capsid surface and a host cellular surface, and the penetration of a virus into a host cell. However, viruses are not directly visible because their sizes are at the order of tens of nanometers. The virus images are constructed from virus information, which is either collected from modalities described above or generated by computer simulations. Therefore, surface/image construction is a part of the virus visualization. Yu and Bajaj present a computational algorithm to segment asymmetric units of three-dimensional (3D) density maps of icosahedral viruses [9] and a computational approach to structural interpretation from reconstructed 3D electron microscopy (3D-EM) maps of viruses [10]. Some basic biomolecular surface methods are available in visualization software packages Chimera (http://www.cgl.ucsf.edu/chimera/) and VMD (http://www.ks.uiuc.edu/Research/vmd/).

The difficulty of characterizing a virus complex is not only its massive number of atoms, or data sets, but also its everlasting interactions. Except for envelope type of viruses which typically cover their capsids by envelopes derived from lipids and proteins of their host cell membranes, most viruses use their own capsids to interact with the environment and host cells. A viral capsid usually consists of many identical viral protein subunits that form the capsid by symmetric assembly. There are strong interactions between viral protein subunits so that viral capsids are rigid enough to hold viral genome material and protect its content. Viruses have adapted a number of strategies to maintain the stability and flexibility of viral capsids. For many small viruses, such as one of STMV, their subunit proteins generally only touch each other by their edges. Their capsid stability is achieved by strong nonbonding interactions (i.e., hydrogen bonding and van der Waals interactions) between edges of subunit proteins. Some large viruses, such as BMV, have developed overlapping strategies to increase the capsid stability. Some viruses even use a few intricately intertwining layers to strengthen their capsids [11]. Virus capsids are further stabilized by their hydrophobic interaction with the aquatic environment. Clearly the boundary profile of the virus complex is determined by the balance of all mechanical forces or equivalently, the energy minimization of the system.

One of the present authors, Wei, introduced some of the first high-order geometric flow equations for image analysis [12]. These equations have led to many applications [12–16]. Mathematical analysis of Wei's equations has been recently carried out in Sobolev space *H* ^1^ by Bertozzi and Greer [17–19], who proved the existence and uniqueness of the solution to a case with *H* ^1^ initial data and a regularized operator. Coupled geometric flow equations were introduced by Wei and Jia for image edge detection [13]. An evolution operator based single-step method was proposed by Wei, Wang and their coworkers for image processing [14]. A partial differential equation approach of Connolly surfaces was proposed by Wei and his coworkers [20]. In such an approach, geometric partial differential equation (PDE) is used to describe the solvent density flows. Most biological processes occur in water, which consists of about 70% body mass. Therefore, in general, the biomolecular surface morphology should be determined by the free energy minimization in the aquatic environment. Wei and his coworkers have addressed this question by considering a mean curvature flow model of bimolecular surfaces that minimize the surface-free energy functional [21]. They have also recently introduced stochastic geometric flows to account for the random fluctuation and dissipation in density and pressure near the surface [22]. A general geometric flow structure, the potential driven geometric flows, was introduced [22]. Physical properties, such as free energy minimization (area decreasing) and incompressibility (volume preserving), were realized in new geometric evolution equations [22]. Computational techniques used in this surface analysis are quite similar to the level sets devised by Osher and Sethian [23–25]. Another efficient approach is the Euler-Lagrange formulation of surface variation developed by Chan and others, [26, 27]. Interacting particle systems and point-based approaches have also been proposed for the modeling and animation of surfaces [28].

An unsolved problem in structural virology is the detailed molecular mechanism of the assembly of virus capsids with the right size that is able to accommodate virus genetic material in the subsequent virus DNA/RNA packaging. Additionally, the process of virus attachment on its host cell, the movement of virus fusion with cellular membrane, and the dynamics of virus penetration into its host cell remain unrevealed mysteries. Prerequisites to unveiling these mysteries are efficient computer science and mathematical tools for modeling virus surface construction, evolution, and visualization, and for analyzing the virus interactions with its host cell. A typical virus has millions of atoms, while a large virus may have tens of millions atoms. Huge viral data sets pose severe challenges to the theoretical understanding and prediction of virus dynamics and interactions. These challenges are considerably exacerbated by the fact that virus behavior and infectivity depend strongly on the physiological environment, where the water molecules are the most common media. This dramatically increases the number of degrees of freedom of a virus system. The real-time dynamic visualization of viral attachment, fusion, and penetration of a host cell in the aquatic environment requires microsecond or even millisecond simulation time and is technically intractable with full-atom models at present [11, 29]. In fact, the elementary operations, that is, the construction of virus surfaces with physical models and real-time visualization of virus morphology present formidable challenges for applied mathematics and computer science.

Recently, one of the present authors, Wei, has developed a differential geometry-based multiscale paradigm to address some of the aforementioned challenges in the nonequilibrium dynamics of viruses, as well as other complex chemical systems, for example, fuel/solar cells, and biological systems, for example, ion channels [30]. In this approach, the differential geometry theory of surfaces and geometric measure theory are employed to couple the macroscopic continuum mechanical description of the aquatic environment with the microscopic discrete atomistic description of the macromolecule. Multiscale action functionals are constructed as a unified framework to derive the governing equations for the dynamics of different scales and different descriptions. The generalized Navier-Stokes equation for the fluid dynamics, the generalized Poisson Boltzmann equation for electrostatic interactions, and Newton's equation for the molecular dynamics were derived by the least action principle. These equations are coupled through the micro-macro boundary whose dynamics is governed by potential driven geometric flows.

The objective of the present work is threefold. First, we apply the differential geometry-based multiscale models to the formation and evolution virus capsids where challenges originated from a large number of atoms and a variety of interactions in a virus system, including the aquatic environment. To dramatically reduce the number of degrees of freedom of a virus system, we treat the water molecules as a macroscopic continuum. However, we maintain atomic description of the virus to allow an optimal access to detailed biomolecular information. Secondly, we propose a new scale, the coarse-grained particles, to improve the earlier multiscale formalism [30]. Our new coarse-grained scale is based on the description of amino acid residues. This additional scale is necessary for excessively large viruses or macromolecules. It efficiently reduces the number of degrees of freedom. Finally, to further reduce computational cost, we utilize virus symmetries to provide an optional reduction in data size. Viruses typically have a few coding genes and they make use of symmetries to reduce their genome size, because capsid genes are repeatedly used. Apparently, viruses also try to make use of symmetry so that they have a high ratio of volume over surface area. As such, virus can maintain the desirable mechanical and chemical stability while without their own cell membranes and complex defense systems. Some of the proposed ideas are tested by their applications in virus surface formation, evolution, and visualization.

## 2. Theory and Algorithms

In this section, differential geometry theory of surfaces and potential driven geometric flows are utilized to establish a multiscale paradigm for modeling and simulation of virus formation and evolution. Then, a coarse-grained virus model is formulated to further reduce the number of degrees of freedom. Finally, the use of symmetry in virus surface construction is discussed.

### 2.1. Differential Geometry-Based Multiscale Model

#### 2.1.1. Multiscale Models of Virus Surface Formation and Evolution

A fundamental issue in biological modeling, and in data analysis, visualization, and dynamical representation is how to deal with a tremendously large number of degrees of freedom resulting from various interaction. Under physiological condition, a virus and its interacting environment may involve tens of millions of protein atoms and water molecules. In principle, the system can be described entirely in the microscopic scale, that is, atomistic description or more detailed description of electrons and nuclei. However, such an approach cannot be productive and does not provide theoretical predictions of physical properties of the virus complex. It is impossible at present, and formidably expensive in near future to describe in full-atomic detail of all the aforementioned interactions for a large virus system. On the other hand, a macroscopic description of the system is incapable of revealing the molecular and atomic information of the virus particle and its dynamics. We plan to reduce the number of degrees of freedom of the virus complex by a differential geometry-based multiscale model. In our multiscale model, we will describe the aquatic environment by a hydrodynamic continuum, that is, a macroscopic description. As such, we are able to dramatically reduce the number of degrees of freedom of millions surrounding water molecules. However, since the biomolecule or the virus is the objective of interest, we will describe the virus in atomic detail, that is, a microscopic, discrete description. Additionally, we carefully consider the solvation process of the virus molecule. The virus surface tension and mechanical work of virus immersion into the solvent are considered in our model, in addition to the possible interaction between virus atoms and the aquatic environment. Finally, the force resulted from virus and solvent interactions is accounted by fluid motion, which is modeled by a viscous fluid.

In our differential geometry-based multiscale model, we use a hypersurface (characteristic) function *S* to characterize the boundary of the virus and solvent. As such, *S* = 1 indicates the virus domain and *S* = 0 (i.e., 1 − *S* = 1) indicates the aquatic domain. However, at atomic scale, the virus surface, or the flow boundary between the virus particle and aquatic environment cannot behave like the Heaviside function. Instead, it must take a value between zero and one (0 ≤ *S* ≤ 1). Such a profile characterizes the boundary between the virus and the aquatic environment. In the rest of this section, we set **x** ∈ ℝ^3^ as the macroscopic variable and **z** = (**z**_1_, **z**_2_,…, **z**_*N*_) ∈ ℝ^3*N*^ as the microscopic variable of *N* discrete atoms or particles. The domain of the solvent is denoted as Ω_*m*_:{**x** | *S*(**x**) ≠ 0} and that of the virus molecule is denoted as Ω_*s*_ : {**x** | (1 − *S*(**x**)) ≠ 0}. The whole computational domain is Ω = Ω_*s*_ ∪ Ω_*m*_. The solvent-solute boundary is Ω_*b*_ = Ω_*s*_∩Ω_*m*_.

We consider the total action functional for the virus complex [30]

(1) $$\begin{array}{cc} & {𝒮}_{\text{total}}\left[S,\phi ,x,z\right]\\ & =∭\{\left[\gamma ||\nabla S||+Sp+\left(1-S\right)\rho_{s}u\right]+S\left[\rho_{m}\phi -\frac{\epsilon_{m}}{2}{\left|\nabla \phi \right|}^{2}\right]\\ & \;\;\;+\left(1-S\right)\left[-\frac{\epsilon_{s}}{2}{\left|\nabla \phi \right|}^{2}-k_{B}T\overset{N_{c}}{\underset{j=1}{\sum }}c_{j}\left({\text{e}}^{-q_{j}\phi /k_{B}T}-1\right)\right]\\ & \;\;\;-\left(1-S\right)\left[\rho_{s}\frac{v^{2}}{2}-p+\frac{\mu_{f}}{8}\int_{}^{t}{\left[\nabla v+{\left(\nabla v\right)}^{T}\right]}^{2}dt'\right]\\ & \;\;\;-S\overset{N}{\underset{j=1}{\sum }}\left[\rho_{j}\frac{{\dot{z}}_{j}^{2}}{2}-U\left(z\right)\right]\}dxdzdt, \end{array}$$

where *ϕ* ∈ Ω is the electrostatic potential, *γ* is the surface tension, *p* is the pressure, *u* is the interaction potential between the solvent and the solute, *k*_*B*_ is the Boltzmann constant, *T* is the temperature, *c*_*j*_ is the bulk concentration of *j*th ionic species, *N*_*c*_ is the number of ionic species, and *ρ*_*m*_(**x**, **z**) = ∑_*j*_ *Q*_*j*_ *δ*(**x** − **z**_*j*_) is the canonical density of molecular free charges, with *Q*_*j*_ being partial charges on (discrete) atoms. Here, *ϵ*_*m*_ = *ϵ*_0_ *ε*_*m*_ and *ϵ*_*s*_ = *ϵ*_0_ *ε*_*s*_ are the permittivities of the macromolecule and the solvent, respectively, where *ϵ*_0_ is the permittivity of vacuum, and *ε*_*α*_ and (*α* = *m*, *s*) are relative permittivities. We treat *ε*_*α*_ as constants. Additionally, *ρ*_*s*_ and *ρ*_*j*_ are mass densities of the solvent and virus atom (or coarse-grained particle), respectively. Finally, **v** ∈ Ω_*s*_ is the fluid velocity, *μ*_*f*_ is the viscosity of the fluid, symbol *T* in superscript denotes the transpose, ${\dot{z}}_{j}=dz_{j}/dt\in \Omega_{m}$ is the velocity of the *j*th atom, and *U* is the interaction potential for atoms.

On the right hand side of (1), the first row is the nonpolar solvation free energy, which includes the surface area effect (*γ*∥∇*S*∥), the mechanical work (the volume effect *S* *p*), and the solvent-solute interactions ((1 − *S*)*ρ*_*s*_ *u*). In principle, these interactions take care of important dispersion effects, and other van der Waals effects. Geometric measure theory is used to come up with the expression for the surface area. The second row is the electrostatic polar solvation free energy, which has contributions from the virus particle *S*[*ρ*_*m*_ *ϕ* − *ϵ*_*m*_/2 | ∇*ϕ* | ^2^] and the aquatic solvent (1 − *S*)[−(*ϵ*_*s*_/2) | ∇*ϕ* | ^2^ − *k*_*B*_ *T*∑_*j*_^*N*_*c*_^ *c*_*j*_(e^−*q*_*j*_ *ϕ*/*k*_*B*_ *T*^ − 1)]. Here, the virus particles contribute a set of discrete partial charges while the ion charges in the solvent are treated as a continuous Boltzmann distribution. This is valid as long as the system is near equilibrium. For systems far from equilibrium, alternative models, such as Poisson-Nernst-Planck (PNP) equations, are required to describe the density of ionic species [30]. The third row is the Lagrangian of the fluid dynamics subsystem with a negative sign. It consists of the kinetic energy (*ρ*_*s*_(**v** ^2^/2)) of the fluid flow and the generalized potential energy. The latter includes pressure (*p*) and stress energy ((*μ*_*f*_/8)∫^*t*^[∇**v**+(∇**v**)^*T*^]^2^ *d* *t*′). The stress energy represents the energy loss due to the interactions among the fluid particles, which are not explicitly described in the present model. The exact expression of the stress tensor for real fluid is usually unknown. Newtonian fluid and NonNewtonian fluid approximations are commonly used, in addition to numerous other approximations. Finally the last row contains the Lagrangian of the virus molecular dynamics subsystem with a negative sign. It describes the kinetic energy $\rho_{j}({\dot{z}}_{j}^{2}/2)$ and potential energy *U*(**z**). The latter includes all possible potential interactions among virus atoms or coarse-grained particles. We have chosen negative signs for two Lagrangians so that the potential energies have positive signs and are consistent with other potential energies.

#### 2.1.2. Governing Equations for Coupled Fluid Dynamics and Molecular Dynamics

In the present work, we derive four governing equations by employing the principle of the least action to the total action functional (*𝒮*_total_[*S*, *ϕ*, **x**, **z**]) in (1) with respect to four variables (*S*, *ϕ*, **x**, **z**)

(2) $$\begin{array}{cc} & \delta {𝒮}_{\text{total}}\left[S,\phi ,x,z\right]\\ & \;=∭\{\left[\nabla \cdot \epsilon \left(S\right)\phi +S\rho_{m}+\left(1-S\right)\overset{N_{c}}{\underset{j}{\sum }}q_{j}c_{j}{\text{e}}^{-q_{j}\phi /k_{B}T}‍\right]\delta \phi \\ & \;\;\;\;+\{\left[-\nabla \cdot \frac{\gamma \nabla S}{||\nabla S||}+p-\rho_{s}u\right]+\left[\rho_{m}\phi -\frac{\epsilon_{m}}{2}{\left|\nabla \phi \right|}^{2}\right]\\ & \;\;\;\;\;-\left[-\frac{\epsilon_{s}}{2}{\left|\nabla \phi \right|}^{2}-k_{B}T\overset{N_{c}}{\underset{j}{\sum }}c_{j}\left({\text{e}}^{-q_{j}\phi /k_{B}T}-1\right)‍\right]\\ & \;\;\;\;\;-\underset{j}{\sum }\left[\rho_{j}\frac{{\dot{z}}_{j}^{2}}{2}-U\left(z\right)\right]\\ & \;\;\;\;\;\left[\rho_{s}\frac{v^{2}}{2}-p+\frac{\mu_{f}}{8}\int_{}^{t}{\left[\nabla v+{\left(\nabla v\right)}^{T}\right]}^{2}dt^{\prime }\right]\}\delta S\\ & \;\;\;\;+[S\nabla p+\left(1-S\right)\nabla \rho_{s}u+S\phi \nabla \rho_{m}+\left(1-S\right)\rho_{s}\\ & \;\;\;\;\;\left(\frac{\partial v}{\partial t}+v\cdot \nabla v\right)+\left(1-S\right)\nabla p-\nabla \cdot \left(1-S\right)𝕋]\cdot \delta x\\ & \;\;\;\;+\underset{j}{\sum }\left[\left(1-S\right)\nabla_{j}\rho_{s}u+S\phi \nabla_{j}\rho_{m}+S\rho_{j}{\ddot{z}}_{j}+S\nabla_{j}U\left(z\right)\right]‍\\ & \;\;\;\;\cdot \delta z_{j}\}dxdzdt=0. \end{array}$$

Here, *δ* *S*, *δ* *ϕ*, *δ* **x**, and *δ* **z** are four infinitesimally small but nonzero perturbations. In order for the first variation to vanish, the terms associated *δ* *S*, *δ* *ϕ*, *δ* **x**, and *δ* **z** have to vanish independently. First, the term associated with *δ* *ϕ* gives rise to a generalized Poisson-Boltzmann equation

(3) $$\begin{array}{c} -\nabla \cdot \epsilon \left(S\right)\nabla \phi =S\rho_{m}+\left(1-S\right)\overset{N_{c}}{\underset{j}{\sum }}q_{j}c_{j}{\text{e}}^{-q_{j}\phi /k_{B}T}‍, \end{array}$$

where

(4) $$\begin{array}{c} \epsilon \left(S\right)=S\epsilon_{m}+\left(1-S\right)\epsilon_{s} \end{array}$$

provides a smooth dielectric profile near the interface. This is a new Poisson-Boltzmann equation for overlapping domains. With the sharp interface, limit, (3) reduces to the standard Poisson-Boltzmann equation [31–35]

(5) $$\begin{array}{c} -\epsilon_{m}\nabla^{2}\phi_{m}=\rho_{m},\;\forall x\in \Omega_{m},\\ -\epsilon_{s}\nabla^{2}\phi_{s}=\overset{N_{c}}{\underset{j}{\sum }}q_{j}c_{j}{\text{e}}^{-q_{j}\phi_{s}/k_{B}T}‍,\;\forall x\in \Omega_{s}, \end{array}$$

and appropriate interface conditions

(6) $$\begin{array}{c} \phi_{s}=\phi_{m},\;\;\epsilon_{m}\nabla \phi_{m}\cdot n=\epsilon_{s}\nabla \phi_{s}\cdot n,\;\forall x\;\text{on}\;\Gamma \\ , \end{array}$$

where Ω_*m*_ and Ω_*s*_ are, respectively, the virus domain and the solvent domain, Γ is the sharp interface and **n** is the normal vector of the surface.

Additionally, the virus surface evolution equation can be constructed by requiring the term associated with *δ* *S* in (1) to vanish, followed by the use of the steepest descent scheme

(7) $$\begin{array}{cc} \frac{\partial S}{\partial t} & =||\nabla S||\{\nabla \cdot \frac{\gamma \nabla S}{||\nabla S||}-p+\rho_{s}u-\rho_{m}\phi +\frac{\epsilon_{m}}{2}{\left|\nabla \phi \right|}^{2}\\ & \;\;\;\;-\frac{\epsilon_{s}}{2}{\left|\nabla \phi \right|}^{2}-k_{B}T\overset{N_{c}}{\underset{j}{\sum }}c_{j}({\text{e}}^{-q_{j}\phi /k_{B}T}-1‍)\\ & \;\;\;\;-\left[\rho_{s}\frac{v^{2}}{2}-p+\frac{\mu_{f}}{8}\int_{}^{t}{\left[\nabla v+{\left(\nabla v\right)}^{T}\right]}^{2}dt'\right]\\ & \;\;\;\;+\underset{j}{\sum }\left[\rho_{j}\frac{{\dot{z}}_{j}^{2}}{2}-U\left(z\right)\right]‍\}. \end{array}$$

The structure of this equation is very similar to the potential driven geometric flows introduced in the earlier work [22, 30]

(8) $$\begin{array}{cc} \frac{\partial S}{\partial t} & =||\nabla S||\left[\nabla \cdot \frac{\gamma \nabla S}{||\nabla S||}+V\right], \end{array}$$

where *V* includes appropriate potential interaction terms. Therefore, (7) can be solved by using the same procedure as that described in the earlier work [22].

Moreover, the requirement of the vanishing of the term associated with *δ* **x** gives rise to a generalized Navier-Stokes equation for continuum fluid dynamics [30]

(9) $$\begin{array}{c} \rho_{s}\left(\frac{\partial v}{\partial t}+v\cdot \nabla v\right)=-\nabla p+\frac{1}{1-S}\nabla \cdot \left(1-S\right)𝕋+F, \end{array}$$

where the stress tensor is given by

(10) $$\begin{array}{c} 𝕋=\frac{\mu_{f}}{2}\left[\nabla v+{\left(\nabla v\right)}^{T}\right]. \end{array}$$

The Newtonian fluid is assumed in the present work. The force in (9) is given by

(11) $$\begin{array}{c} F=\frac{S}{1-S}f. \end{array}$$

Here, the force includes a few components

(12) $$\begin{array}{c} f=f_{\text{P}}+f_{\text{SSI}}+f_{\text{RF}}, \end{array}$$

defined as

(13) $$\begin{array}{cc} & f_{\text{P}}=-\nabla p;\\ & f_{\text{SSI}}=-\frac{\left(1-S\right)}{S}\nabla \left(\rho_{s}u\right),\\ & f_{\text{RF}}=\frac{\rho_{m}}{S}\nabla \left(S\phi \right). \end{array}$$

The detailed derivation of the generalized Navier-Stokes equation can be found in [30]. In case of sharp solvent-virus interfaces, the hypersurface function *S* becomes a step function, and (9) reduces to the standard Navier-Stokes equation

(14) $$\begin{array}{c} \rho_{s}\left(\frac{\partial v}{\partial t}+v\cdot \nabla v\right)=-\nabla p+\mu_{f}\nabla^{2}v+F, \end{array}$$

with simplified force expressions.

Finally, the Newton's equation for molecular dynamics of the *j*th atom (particle) in the virus is derived from the term associated with *δ* **z**_*j*_,

(15) $$\begin{array}{c} \rho_{j}{\ddot{z}}_{j}=f^{j}. \end{array}$$

Here the microscopic force associated with the *j*th atom is

(16) $$\begin{array}{c} f^{j}=f_{\text{SSI}}^{j}+f_{\text{RF}}^{j}+f_{\text{PI}}^{j}. \end{array}$$

The force components are defined as

(17) $$\begin{array}{cc} & f_{\text{SSI}}^{j}=-\frac{\left(1-S\right)}{S}\nabla_{j}\left(\rho_{s}u\right)\\ & f_{\text{RF}}^{j}=\frac{\rho_{m}}{S}\nabla_{j}\left(S\phi \right)\\ & f_{\text{PI}}^{j}=-\nabla_{j}U\left(z\right), \end{array}$$

where **f**_SSI_^*j*^, **f**_RF_^*j*^, and **f**_PI_^*j*^ are, respectively, solvent-solute interaction force, reaction field force, and potential interaction force.

In this multiscale system, all forces are balanced. The fluid dynamics, the molecular dynamics, the electrostatic subsystem, and the hypersurface function are all coupled.

### 2.2. Coarse-Grained Model

**Figure 1:**
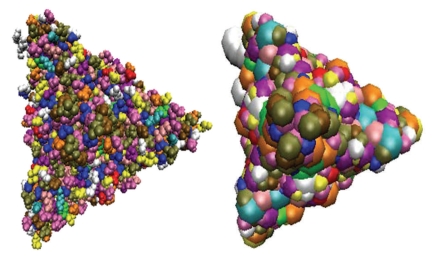
Coarse-grained model of a viral protein subunit. Left: the full atomic model of a protein subunit of the Nodamura virus (PDB ID: 1nov), Right: the coarse-grained model of a protein subunit of the Nodamura virus.

 As a part of our multiscale framework, we consider a coarse-grained formalism for viral surface formation and evolution. Coarse-grained models are often used to deal with exceptionally large biological systems. In the present treatment, we consider each amino acid residue as a particle, located at the C_*α*_ position. The radii of twenty standard amino acid residues used in the present work are listed in Table 1. Coarse-grained representations are efficient approaches for data size reduction. Combined with enhanced computer power and efficient computational algorithms, coarse-grained approaches currently enable the simulation of systems of biologically relevant size (submicrometric) and timescale (microsecond or millisecond) [29]. Although coarse-grained models cannot be considered as predictive as all-atom ones, they can provide much insight with the use of more rigorous parameterization techniques and efficient algorithms for sampling configurational space. Since the simulation size and timescale of coarse-grained models coincide with those that can be reached with the most advanced spectroscopic techniques, it is possible to directly compare experiment data and simulation predictions. In this work, we will explore the use of coarse-grained models for viral surface formation and evolution. Figure 1 presents an illustration of coarse-graining particles for a viral protein subunit. The original full-atomic subunit of the Nodamura virus has about 10 thousand atoms. In the coarse grained representation, each amino acid residue is considered as one particle, located at the position of the original C_*α*_ atom. Each type of amino acid residues has a particle radius as shown in Table 1. The discrete-continuum model of viral surface representation discussed above is still applicable to the present coarse-grain-continuum setting. However, to use (7) for viral surface formation and evolution, we need to redefine the Lennard-Jones and Coulomb potential parameters to describe the interaction between amino-acid-residue particles.

### 2.3. Viral Data Size Reduction by Symmetry

#### 2.3.1. Symmetry in Virus Capsids

**Figure 2:**
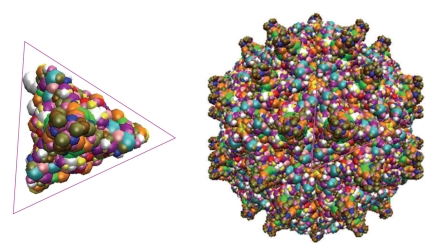
Illustration of surface construction from a facet patch by using symmetry. Left: the generating subunit (facet patch) of the Nodamura virus (PDB ID: 1nov), Right: the full surface of the Nodamura virus constructed by symmetric assembly.

Viral data may involve tens of millions of atomic coordinates and radii, and are enormously large for structural modeling, simulation and visualization. Viral dynamical cycles may last from millisecond to days, and real-time full-atom viral dynamical simulations of viruses are intractable to the present computational capability [11]. However, viruses typically have very small genomes and code a few proteins. In order for a viral capsid to hold all viral genetic material, virus makes use of symmetry in its capsid assembly. Amazingly, most viruses are symmetric, having icosahedral, helical, dihedral, or circular symmetries [6]. As such, an icosahedral virus can self-organize one protein to generate a capsid of 60 symmetry-related subunits (some viruses code hundreds of proteins). Therefore, it is desirable to take advantage of symmetry in viral data analysis, operation, and management. In particular, we propose to make use of viral symmetries, if they are available in our geometric flow based viral surface formation and evolution. Additionally, we can detect partial and approximate symmetry [36] from viral surfaces, and enforce symmetrization [37]. As such, we will use geometric flows to generate symmetric facets, or patches from viral protein subunits, and construct the whole viral surface by symmetric assembly of viral facets; see Figure 2 for an illustration.

#### 2.3.2. Virus Symmetry Transformation

 Viruses have adapted five point group symmetries, that is, circular, dihedral, tetrahedral, octahedral, and icosahedral, in their biological assemblies. Mathematically, only three types of symmetric operations, that is, rotation, inversion, and translation are involved. Starting with the basic set of coordinates of a protein subunit, the virus capsid data can be obtained by the transformation

(18) $$\left(\begin{array}{c} X\\ Y\\ Z \end{array}\right)=\left(\begin{array}{cccc} r_{11} & r_{12} & r_{13} & t_{1}\\ r_{21} & r_{22} & r_{23} & t_{2}\\ r_{31} & r_{32} & r_{33} & t_{3} \end{array}\right)\left(\begin{array}{c} x\\ y\\ z\\ 1 \end{array}\right),$$

where *r*_*i* *j*_ are rotational (or inversion) elements, and *t*_*i*_ are translational elements. The viral data deposited in the Protein Data Bank (PDB) often have problems with missing sets of transformation operations and erroneous coordinate-frame representations. We make corrections by using the Virus Particle Explorer database [7] (VIPERdb; http://viperdb.scripps.edu/) and/or the Protein Quaternary Structure server (PQS; http://pqs.ebi.ac.uk/).

## 3. Numerical Demonstration

Recent advances in structural biology and microbiology have given rise to an increasing body of structural data for over 300 viruses and viral complexes. Quaternary structures of viruses and viral complexes pose many challenges for viral representation, visualization, and the analysis of virus stability and interaction [6]. The proposed multiscale framework can be studied on a wide range of test cases to demonstrate its utility and usefulness to the research community. However, a full-scale demonstration of the proposed multiscale model is a rather computationally challenging task as it involves computational fluid dynamics (CFD), molecular dynamics (MD) of viruses, and surface dynamics of large systems. In this paper, we should primarily focus on the virus surface formation and evolution. The coupling of the surface dynamics to the CFD and MD will be studied in our future work and published elsewhere.

**Figure 3:**
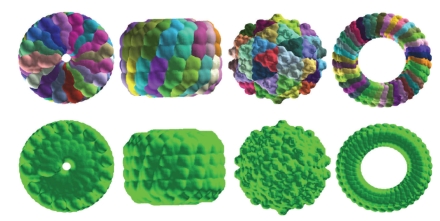
Illustration of virus surfaces constructed by using the proposed geometric flow approach in conjunction with the coarse-grained model and the symmetry assembly. Upper row: Surfaces generated from a facet patch by using symmetry assembly. Lower row: Surfaces generated without the use of symmetry. From left to right: Cucumber green mottle mosaic virus (CGMMV) with helical symmetry (1cgm), Tobacco mosaic virus coat protein four-layer aggregate with D_17_ symmetry (1ei7), Nodamura virus with icosahedral symmetry (1nov), and Viral toxin pneumolysin with C_38_ circular symmetry (2bk1).

We also test two other proposed ideas in this work, that is, the coarse-grained virus model and the use of symmetry assembly for the virus surface construction. In particular, we are interested in examining the effect of the symmetry assembly on the virus surface visualization. As shown in Figure 3, we consider the coarse-grained model, which is an efficient way to reduce computational cost. Additionally, we test the surface construction by using symmetric assembly. In comparison with surfaces constructed by potential driven geometric flows without using the symmetry (Lower row), geometric flow surfaces constructed by symmetry (Upper row) provide a good representation of the original surfaces. However, one can still see that contact edges in the surfaces constructed by symmetry are not very smooth. Moreover, we expect some impact of symmetric assembly to the MD and fluid dynamics, as the symmetry becomes an additional constraint to virus dynamical motions. The soundness of such a constraint needs to be studied. This aspect as well as many other ideas proposed in this work will be further explored elsewhere.

## 4. Concluding Remarks

The control of infective viruses released by terrorists, and the prevention of viral epidemics and pandemics, such as HIV, SARS, H1N1, and bird flu are of tremendous importance. The understanding of viral surface formation, evolution, viral attachment and penetration of host cells are prerequisites to viral disease prevention and control. This problem, as well as many other similar problems in molecular biology, poses pressing challenges to the theoretical community due to their large number of degrees of freedom. The main purpose of the present work is to introduce a differential geometry-based multiscale framework to handle complex biological systems. The present multiscale model couples macroscopic fluid dynamics, microscopic molecular dynamics, and surface dynamics in a unified framework. The differential geometry theory of surfaces is utilized to put continuum description and discrete description in an equal footing. The present work constructs a generalized action functional to self-consistently couple different scales. Governing equations for the fluid dynamics, that is, the generalized Navier-Stokes equation, and molecular dynamics, that is, the Newton's equation, are derived by minimizing the action functional. Additionally, we make use of viral symmetry to dramatically reduce viral data sizes and improve viral visualization. Finally, some of the proposed approaches are demonstrated by the generation of a few virus surfaces.

The proposed differential geometry-based multiscale model can be easily generalized to complex systems with multiple interfaces or many biomolecules. Additionally, the incorporation of continuum solid description into the present model will be published elsewhere. Finally, the inclusion of a quantum mechanical description can also be pursued in a similar way and will be published elsewhere. Numerical experiments that further demonstrate the proposed ideas are under our consideration.

## Figures and Tables

**Table 1:** Coarse-grain radii (Å) for twenty standard amino acid residues.

Residue	Radius	Residue	Radius	Residue	Radius	Residue	Radius	Residue	Radius
GLY	4.20	ALA	4.10	VAL	4.30	LEU	5.70	ILE	5.60
PRO	4.10	PHE	7.00	TYR	8.30	TRP	8.30	SER	4.30
THR	4.40	ASN	5.50	GLN	6.80	CYS	4.50	MET	7.30
ASP	5.50	GLU	6.70	HIS	6.40	LYS	8.20	ARG	9.10
